# Structure of the T9SS PorKN ring complex reveals conformational plasticity based on the repurposed FGE fold

**DOI:** 10.1128/mbio.01799-25

**Published:** 2025-08-07

**Authors:** Xiangan Liu, John D. Perpich, Liqiang Song, Christian Cambillau, Thierry Doan, Lei Zheng, Richard J. Lamont, Eric Cascales, Bo Hu

**Affiliations:** 1 Department of Microbiology and Molecular Genetics, McGovern Medical School, Houston, Texas, USA; 2 Department of Oral Immunology and Infectious Diseases, University of Louisville 5170, Louisville, Kentucky, USA; 3 Laboratoire d’Ingénierie des Systèmes Macromoléculaires (LISM, UMR7255), Aix-Marseille Université, CNRS 128791, Marseille, France; 4 School of Microbiology and APC Microbiome Ireland, University College Cork 8795, Cork, County Cork, Ireland; 5 Department of Biochemistry, McGovern Medical School, Houston, Texas, USA; University of Wisconsin-Madison, Madison, Wisconsin, USA

**Keywords:** type 9 protein secretion, surface structures, cryo-EM

## Abstract

The type IX secretion system (T9SS) is a protein secretion machinery unique to the Bacteroidetes-Chlorobi-Fibrobacteres superphylum, which plays crucial roles in bacterial pathogenesis and gliding motility. It is composed of >15 proteins, including the proton-motive force-dependent PorLM motor, the PorKN ring anchored to the outer membrane, and the Sov translocon. Here, we present the cryo-electron microscopy (EM) structure of the PorKN ring complex from *Porphyromonas gingivalis* at 3.2 Å resolution. Our structural analysis reveals that PorK contains a repurposed formylglycine-generating enzyme-like fold, which serves as a structural hub for complex assembly rather than enzymatic activity. The complex exhibits a 33-fold symmetry with PorK and PorN assembling two tightly packed and wedged subrings. The structure reveals previously uncharacterized N- and C-terminal helices in PorN that are crucial for PorK binding and complex stability. By combining our high-resolution structure with *in situ* cryo-electron tomography data, we propose a mechanism whereby PorKN undergoes conformational changes during substrate transport, transitioning between 50° and 90° states relative to the membrane plane. Finally, structural predictions coupled to site-directed disulfide cross-linking identified contacts between PorM and the PorKN ring. Collectively, these findings provide crucial insights into the molecular architecture and dynamic behavior of the T9SS machinery, advancing our understanding of bacterial protein secretion mechanisms.

**IMPORTANCE:**

The bacterial type IX secretion system (T9SS) is essential for processes such as gliding motility and secretion of virulence factors. In *Porphyromonas gingivalis*, a major periodontal pathogen, the T9SS transports over 30 virulence-associated proteins, making it central to disease development. The T9SS core is composed of PorLM motors that are thought to energize the PorKN outer membrane-associated ring. However, the molecular architecture of the PorKN ring has remained unresolved. Here, we present its atomic-resolution cryo-EM structure, revealing a formylglycine-generating enzyme-like fold in PorK that mediates PorK-PorN interactions through specific insertion motifs. Our results show that the ring exhibits intrinsic structural plasticity, including dynamic flexibility and variable stoichiometry. AlphaFold models and disulfide cross-linking experiments further provide information on how PorLM motors are connected to the PorKN ring. These insights redefine our understanding of the T9SS mechanism of action and offer a structural framework for the development of targeted antimicrobial strategies.

## INTRODUCTION

The type IX secretion system (T9SS) represents a distinct bacterial secretion machinery found exclusively within the Bacteroidetes-Chlorobi-Fibrobacteres superphylum (1, 2). This system serves three primary functions across different bacterial species: facilitating protein secretion in pathogenic bacteria such as *Porphyromonas gingivalis*, participating in predation in Aureispira (3), and enabling protein secretion and gliding motility in species such as *Flavobacterium johnsoniae* (4–8). In *P. gingivalis*, the T9SS is essential for virulence, secreting over 30 different proteins, including gingipains, hemagglutinins, and peptidylarginine deiminase, an enzyme potentially linked to rheumatoid arthritis (9–12).

**Fig 1:**
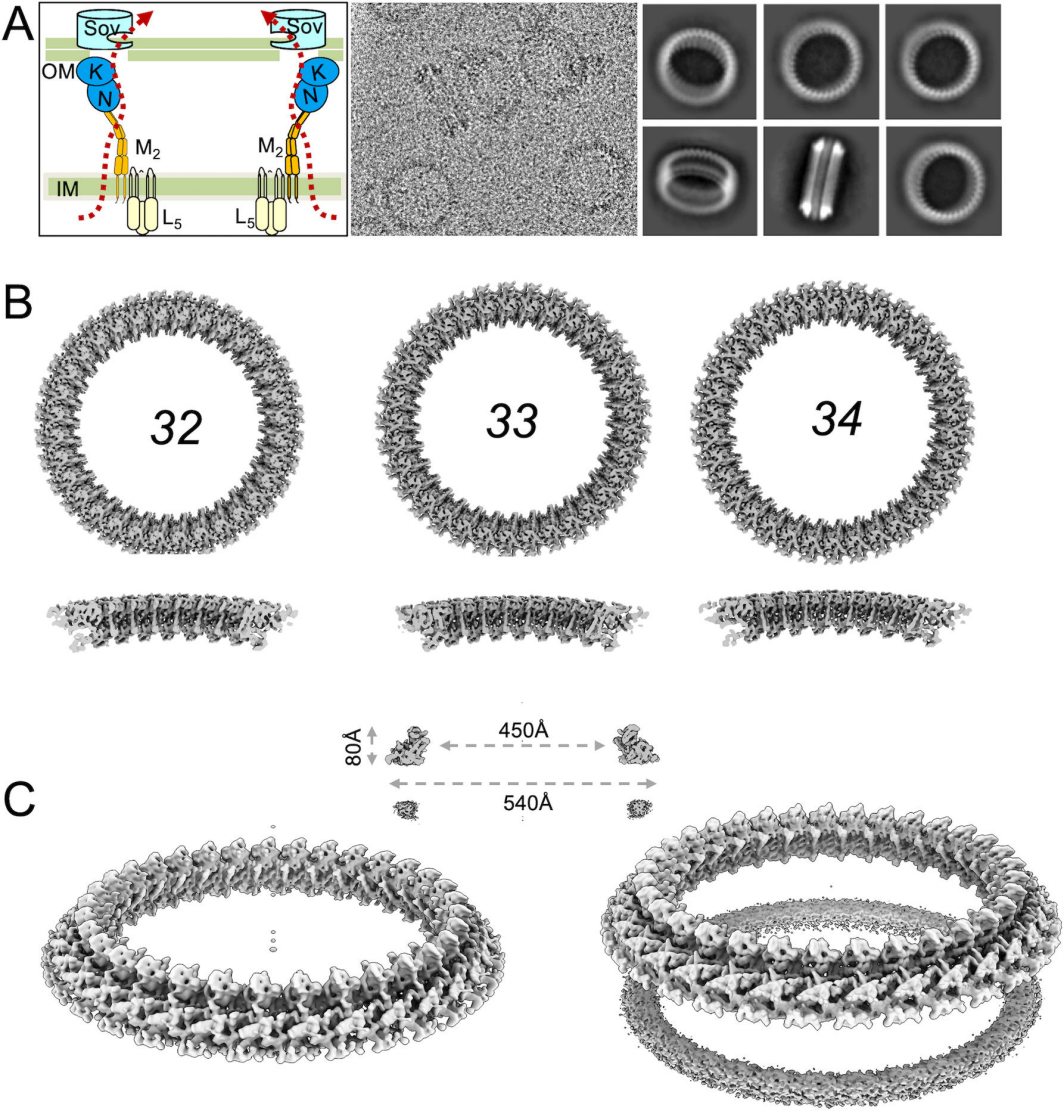
PorKN rings: single and double forms with varying symmetries. (**A**) Schematic of the structure and function of the T9SS of *P. gingivalis* and representative image of PorKN ring particles visualized by cryo-EM. The 2D class averages show single and double rings in various orientations. (**B**) Top views of PorKN rings in 32-, 33-, and 34-fold symmetries. Corresponding cutaway views are shown in the second row. (**C**) Tilted views of single PorKN ring (left) and double ring (right) with 33-fold symmetry. Geometric measurements are displayed in the top-middle section.

The T9SS machinery of *P. gingivalis* comprises approximately 17 proteins that assemble into a large cylindrical, cage-like, complex spanning the bacterial cell envelope (7, 13, 14) (Fig. 1). The system is organized into three core functional modules: the trans-envelope complex, the translocon complex, and the attachment complex (7, 15–19). Within the trans-envelope complex, PorK and PorN form a ~3 MDa ring structure approximately 50 nm in diameter beneath the outer membrane (OM), bridging the proton-motive force (pmf)-dependent PorLM molecular motor and the translocon Sov, thus representing a crucial component of the secretion machinery (14, 20). The PorM dimer and PorL pentamer assemble as a molecular motor in the inner membrane (16, 17, 19, 21, 22) while Sov, the largest β-barrel protein identified in bacteria to date, forms the translocon responsible for substrate translocation across the outer membrane (18, 23). Our previous study indicates that each individual T9SS macromolecular structure resembles a cage in which 18 PorLM motors are connected to the PorKN ring and accommodating eight Sov translocons (14). These features make the T9SS a uniquely organized bacterial secretion system.

T9SS substrates are recognized in the periplasm through a conserved C-terminal domain and are transported to the extracellular milieu medium or the cell surface using the energy of the pmf, where they are processed (10, 18, 19, 21, 23, 24). While the mechanism prior to their translocation through the Sov translocon is not yet known, it has been proposed that T9SS substrates bind to the PorKN ring, whose PorLM- and pmf-dependent rotation distributes them to the translocons (6–8, 13). Indeed, recent *in situ* structural studies indicate that the PorKN complex undergoes dramatic conformational changes during substrate transport, with individual subunits exhibiting coordinated movements driven by the PorLM motor complex (14). Here, we present the high-resolution cryo-EM structure of the PorKN ring complex, revealing unprecedented details of its molecular organization and suggesting a mechanism for substrate transport. While the current models suggest that PorK and PorN function as a track-like structure, with either the whole PorKN ring rotating or the PorN subring rotating along the PorK subring (25), the wedged interconnection of PorK and PorN is incompatible with the proposed rotational model of PorN along PorK. The PorKN ring structure and the predicted association of PorM with it provide crucial insights into the structural basis of T9SS function and on its operational mechanism.

## RESULTS

### Overall structure of PorKN ring

Initially, we followed the protocol described by Gorasia et al. (20) to purify the PorKN rings from *P. gingivalis*, but the yield was low. Thus, we fused a twin strep-tag to the C-terminal of PorK, which did not affect the functionality of the T9SS. Through affinity purification, we were able to enrich sufficient PorKN rings for cryo-EM imaging. The particles were frozen onto carbon film-covered grids from which we collected about 6,000 images and picked 64,500 particles from these images. A representative cryo-EM micrograph and some typical 2D averaged particle images are shown in Fig. 1A. The particles exhibited two distinct configurations: single and double rings. The double ring was also observed in a previous study (20), likely due to hydrophobic interactions between the two rings. We successfully sorted approximately 37,000 high-quality particles from the boxed particle set using CryoSPARC’s 2D and 3D classification. Of these, ~10,000 exhibited a single-ring configuration, while ~27,000 organized as double rings. By imposing 33-fold symmetry, we resolved the maps at 7.4 Å and 5.6 Å resolutions for single and double rings, respectively (Fig. 1C). The ring’s geometry departs from a standard cylindrical form, instead exhibiting a conical layered structure. Dimensional measurements revealed the outer diameters at the top and bottom as 450 Å and 540 Å, with a height of approximately 80 Å (Fig. 1C).

Notably, the ring’s symmetry number remains ambiguous in the 2D class averages due to the blurriness of a small portion of the ring. The 2D averaged images suggest potential 32-, 33-, or 34-fold symmetry, in agreement with previous cryo-EM and cryo-electron tomography (ET) lower resolution structures (14, 20). To assess the most likely configuration, we reconstructed the ring with each of these symmetries. Based on a comparative analysis of map resolutions, the 33-fold symmetry represents the most likely configuration (Fig. 1B). However, definitively determining the exact fold number (32, 33, or 34) remains challenging, primarily due to the inherent structural plasticity of this large ring-shaped complex, and it is possible that 32-, 33-, and 34-fold symmetries co-exist. In comparison to the single-ring structure, the double-ring configuration exhibited better resolution, suggesting that the combination of two rings likely provides reciprocal structural stabilization, which diminishes the intrinsic flexibility characteristic of a standalone single-ring form. Furthermore, the relative position of the two rings is not fixed due to the blurriness of the bottom ring in the reconstructed double-ring map (Fig. 1C).

### Refinement to atomic resolution

**Fig 2:**
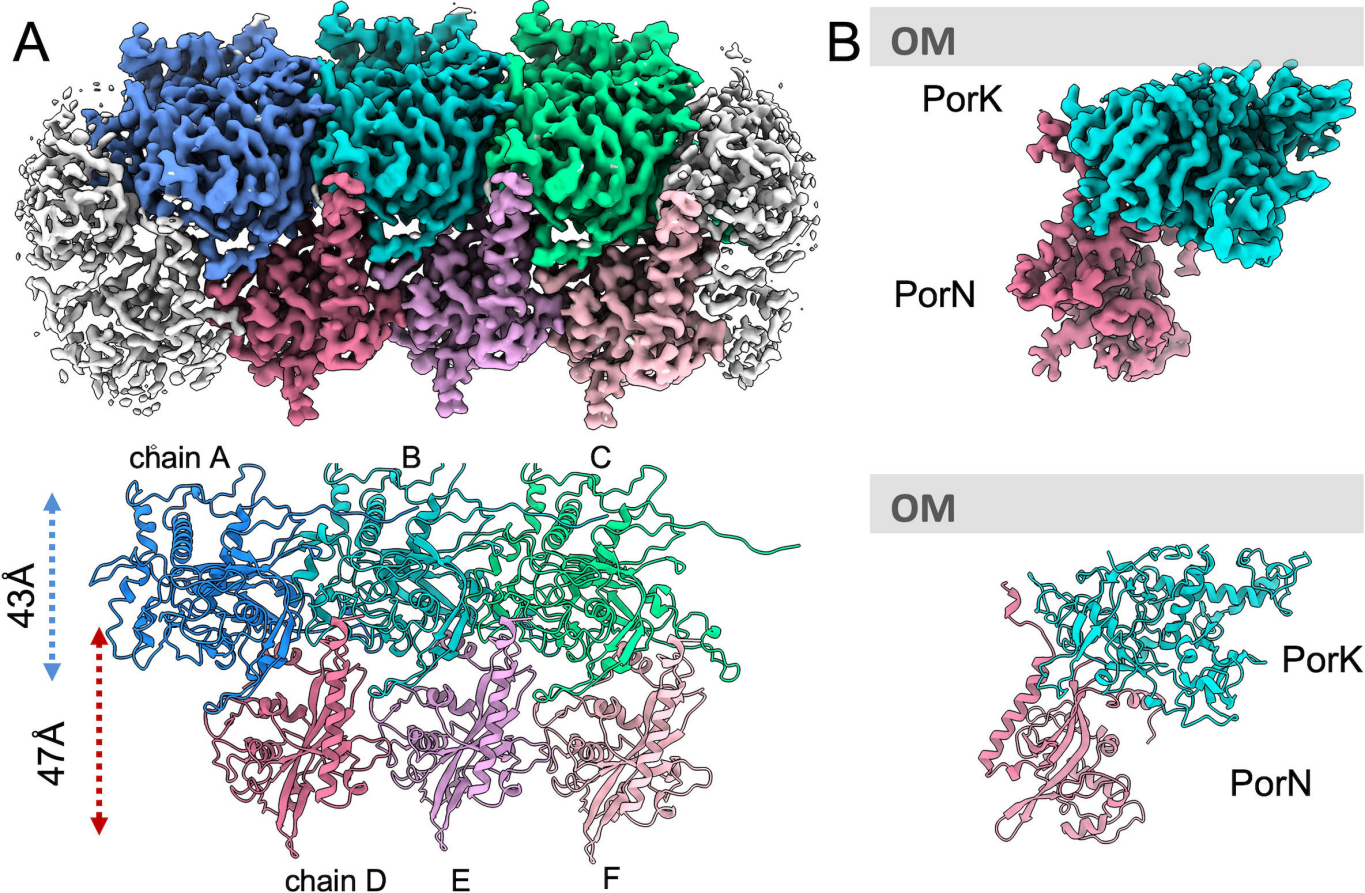
Cryo-EM structure of PorKN ring. (**A**) Upper: focus-refined PorKN ring segment at 3.2 Å resolution, with three dissected subunits highlighted in different colors. Lower: corresponding atomic models of three consecutive PorK and PorN subunits. (**B**) Side view of a single unit of PorK and PorN, along with the corresponding atomic models. The outer membrane’s relative position is indicated.

The reconstructed maps of the entire ring exhibit limited resolution, allowing us to discern only basic helical and beta secondary structure information (Fig. 1B). This resolution limitation likely stems from the ring’s inherent flexibility, which prevents maintenance of a strict symmetry across its entire ring structure. To overcome these limitations, a focused refinement strategy was employed. Initially, approximately six subunits were cropped from the whole ring map as a preliminary model. By expanding the orientation to 33 symmetrized orientations and locally aligning particle images to the cropped ring segment, the resolution was improved significantly. The focused refinement technique enabled the map to reach a 4.2 Å resolution for a segment of six consecutive subunits and further refined to 3.2 Å using only four consecutive subunits (Fig. 2). Notably, the local refinement, performed without imposed symmetry, revealed better-resolved density in the middle portion compared to the regions near the ring’s ends. This asymmetric resolution further confirms the ring’s inherent flexibility and non-rigid nature.

This new map allowed us to trace density connectivity and successfully delineate PorK and PorN subunits. Using the better-resolved middle portion density, we built atomic-resolution structural models for PorK (residues 11–456) and PorN (residues 25–283). We further fitted the Protein Data Bank (PDB) models into their corresponding adjacent density units, labeled as chains A, B, and C for PorK and chains D, E, and F for PorN, as illustrated in Fig. 2A. In the purified ring complex, PorK occupies the wider portion of the ring structure, while PorN stands vertically between neighboring PorK subunits, forming the ring’s narrower portion. Previous studies have determined PorK to be a lipoprotein associated with the OM (16), while the lower part of the PorN structure interacts with the C-terminal D4 domain of the PorM pillar (16, 17). Based on this information and on the location of the N-terminus of PorK, which is acylated and attached to the outer membrane, it is possible to position the PorKN complex underneath the outer membrane (Fig. 2B), in agreement with *in situ* imaging (10). The ring’s total height measures approximately 80 Å, with PorK spanning around 43 Å and PorN extending to about 47 Å. At their interface, an overlapping region of roughly 10 Å in height exists between PorK and PorN. This structural overlap provides a mechanical constraint that stabilizes the interaction between PorK and PorN, potentially limiting their relative circumferential mobility between PorK and PorN rings.

The rotation angles between adjacent subunits using the built PorK and PorN models were 10.857° (PorK chain B to A), 10.864° (B to C), 10.961° (PorN chain E to D), and 10.909° (E to F). These values closely match the theoretical 10.909° rotation for perfect 33-fold symmetry. However, the reconstructed density likely represents an average of mixed 32-, 33-, and 34-fold symmetry configurations. This structural flexibility is supported by differential map-model fitness scores from our PDB deposition: central chains (PorK chain B: 0.436; PorN chain E: 0.409) fit better than peripheral chains (PorK A/C: 0.393/0.397; PorN D/F: 0.370/0.358), indicating conformational heterogeneity within the complex.

### *In vivo* validation of PorKN ring assembly

To test how PorK and PorN are arranged *in vivo*, we used a structural model of PorK_3_-PorN_3_ predicted by AlphaFold2 to identify residues at the PorK-PorK and PorK-PorN interfaces with suitable Cα-Cα distances (<7 Å) and side-chain orientation to be substituted by cysteines. With a root mean square deviation (r.m.s.d.) of 2.7 Å, this AlphaFold2 model appeared later to be almost identical to the cryo-EM structure (Fig. S1). We then predicted structural models of the cysteine mutants to validate the selected sites and to check that the substitutions do not interfere with protein folding or complex formation. Candidate residues at the PorK-PorK and PorK-PorN interfaces were substituted by cysteines using site-directed mutagenesis, and we tested their ability to form disulfide-bond dimers in a reconstituted *in vivo* model of the T9SS PorKLMN trans-envelope complex in the heterologous host *Escherichia coli*.

#### PorK-PorK

**Fig 3:**
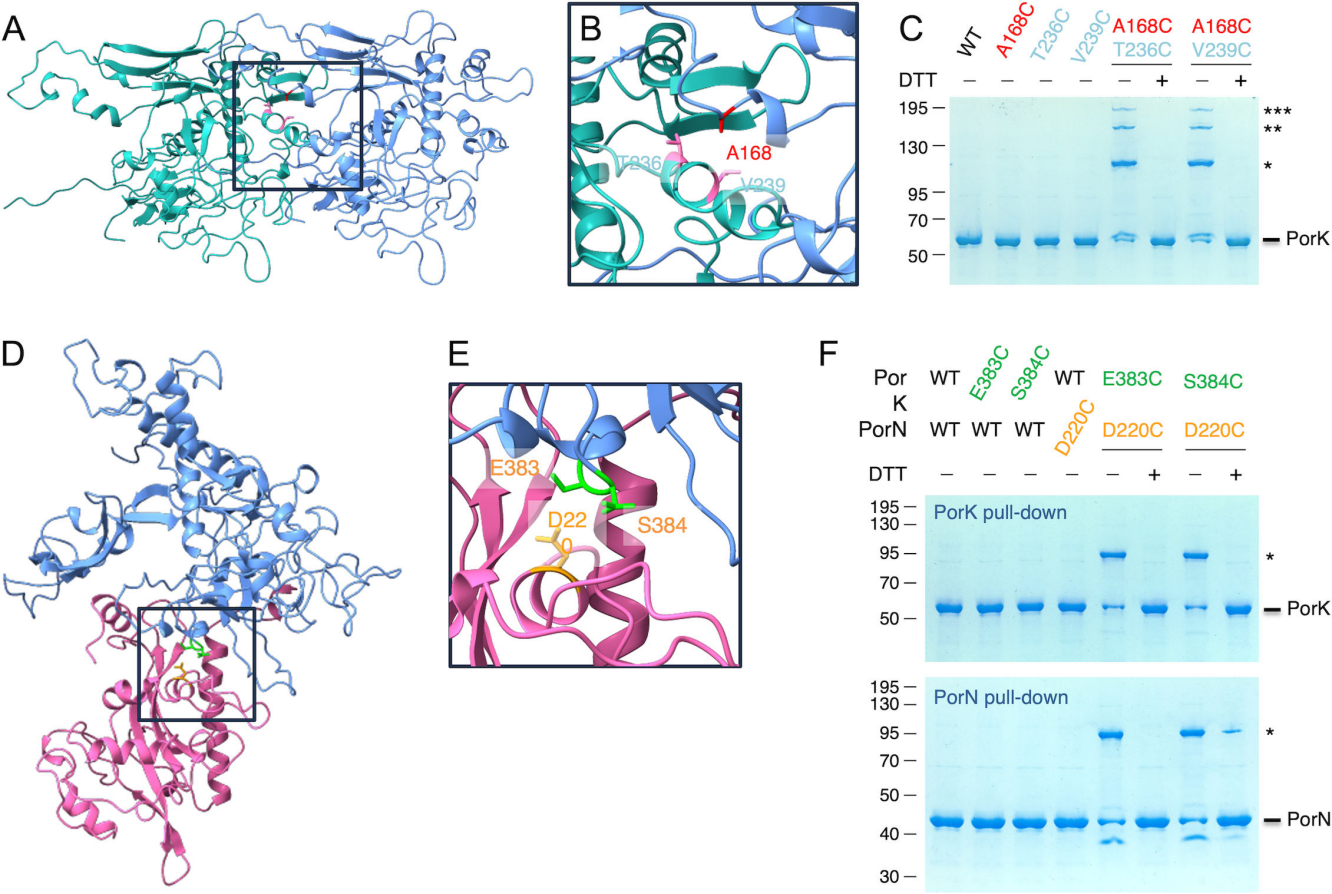
*In vivo* validation of PorK-PorK and PorK-PorN interactions. (**A**) Cryo-EM structure of two contiguous PorK subunits (green and blue). (**B**) Magnification of a portion of the PorK-PorK interface, highlighting the side chains of the substituted residues: T236 and V239 from the “green” monomer (pink), and A168 from the “blue” monomer (red). (**C**) Solubilized membrane extracts of *E. coli* cells producing VSV-G-tagged PorL, FLAG-tagged PorM, Strep-tagged PorN, and 6× His-tagged wild type (WT) or indicated cysteine PorK variants were subjected to pull-down on Ni-NTA resin. The eluate proteins were treated (+) or not (−) with DTT and subjected to 10% acrylamide SDS-PAGE and Coomassie Blue staining. The positions of PorK and PorK multimers (*, **, and ***) are indicated on the right. Molecular weight markers (kDa) are indicated on the left. (**D**) Cryo-EM structure of a PorK-PorN heterodimer (blue and purple, respectively). (**E**) Magnification of a portion of the PorK-PorN interface, highlighting the side chains of the substituted residues: PorK E383 and S384 (green) and PorN D220 (orange). (**F**) Solubilized membrane extracts of *E. coli* cells producing VSV-G-tagged PorL, FLAG-tagged PorM, Strep-tagged WT or cysteine PorN variants, and 6× His-tagged WT or indicated cysteine PorK variants were subjected to pull-down on Ni-NTA (top panel) or StrepTactin (bottom panel) resins. The eluate proteins were treated (+) or not (−) with DTT and subjected to 10% acrylamide SDS-PAGE and Coomassie Blue staining. The positions of PorK, PorN, and PorK-N complex (*) are indicated on the right. Molecular weight markers (kDa) are indicated on the left.

Two pairs of cysteines were introduced at positions A168 and T236, and A168 and V239 (Fig. 3A and B) into the cysteine-less PorK C237S variant, to probe the interface of PorK-PorK subunits within the ring. As shown in Fig. 3C, higher molecular-weight species (denoted with *, **, and ***) were observable for the two A168C/T236C and A168/V239C combinations. The apparent molecular weights of these species (*, ~105 kDa; **, ~150 kDa; ***, ~200 kDa) are compatible with PorK (theoretical mass: 53 kDa without the N-terminal acylation) dimers, trimers, and tetramers. These complexes are likely to be covalently bound by disulfide bridges as they dissociate upon the addition of the reducing agent dithiothreitol (DTT). The presence of only PorK in the three high molecular weight bands (*, **, and ***) was confirmed by mass spectrometry.

#### PorK-PorN 

To test the organization of the PorK and PorN subunits within the ring, we positioned cysteine residues at the interface of the two proteins, as arranged in the cryo-EM structure. The two pairs involve the PorN D220C variant with the PorK E383C or S384C substitutions (Fig. 3D and E). Figure 3F shows that the two pairs are engaged in disulfide-bond formation, as a DTT-sensitive ~90 kDa complex (a weight compatible with a PorK [theoretical mass, 53 kDa] and PorN [theoretical mass, 40 kDa] complex) was pulled down through PorK (Fig. 3F, upper panel) and PorN (Fig. 3F, lower panel). In addition, these bands contain both PorK and PorN as confirmed by mass spectrometry analyzes.

Taken together, the results of the disulfide cross-linking assays demonstrate that PorK and PorN subunits are arranged in the purified ring or in the AlphaFold predicted structure as they are *in vivo*.

### Structure of PorK

**Fig 4:**
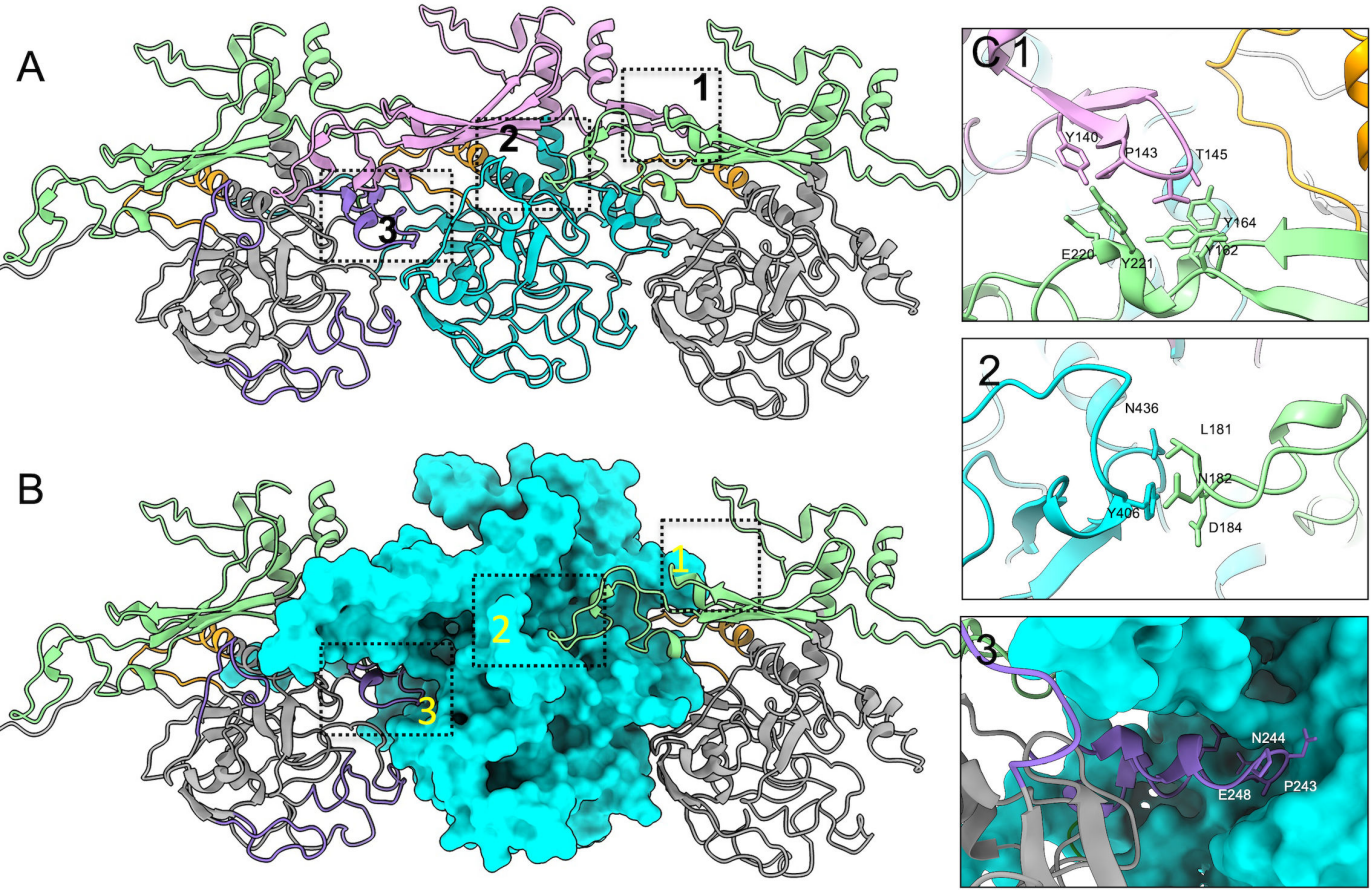
Interactions between PorK subunits. (**A**) Structure of three consecutive PorK subunits, with FGE-like folding domains shown in gray and cyan, the major insertion domain in green and pink, and the minor insertion region and variable regions in orange and purple, respectively. (**B**) The middle PorK subunit is displayed in surface representation. (**C**) Three inter-subunit interaction sites are highlighted within boxed regions.

The PorK structure consists of two key components: a base formylglycine-generating enzyme (FGE)-like fold domain (Fig. 4A, colored in gray and cyan); and two insertion domains, identified through a comprehensive 3D structure search using the DALI server (26). While FGE typically catalyzes the oxidation of cysteine to formylglycine (27), our structural analysis reveals that the FGE-like fold domain in PorK is unlikely to have enzymatic activity due to the absence of the two key cysteine catalytic residues.

Compared to a canonical FGE structure (PDB ID: 6muj), two distinct insertions (Ins) exist in PorK at major helical regions: the horizontal helix (N70-L89) and the vertical helix (W270-G287). The primary insertion, designated InsH, spans residues R81 to P230 (Fig. 4A, colored in pink and green), while a secondary insertion, labeled InsV, is located between residues S281 and F299 (Fig. 4A, colored in orange). The large InsH domain functions as a bridging element for PorK self-association, critically contributing to the PorK ring structure (Fig. 4B). Concurrently, the FGE-like fold domain mediates interactions with PorN, demonstrating a remarkable adaptation of the protein scaffold for protein-protein interaction.

Within the FGE-like fold domain, most structural elements preserve a folding pattern highly similar to FGE, highlighting the conservation of the core scaffold despite its functional transformation. We identified five distinct variable regions (VR): VR1 (G39-S53), VR2 (P233-H257), VR3 (L327-D348), VR4 (L396-K409), and VR5 (K419-I425; Fig. 4A, highlighted in purple in the left PorK unit). In canonical FGE, these regions collectively form a substrate-binding and activation groove. In contrast, PorK’s variable regions now serve as critical interaction interfaces for docking adjacent PorN. This repurposing of the substrate-binding groove into protein-protein interaction interfaces represents a remarkable adaptation of protein architecture.

The repurposed variable regions of the FGE-like domain and insertion domains are deeply involved in the formation of the PorK ring, revealing intricate interaction mechanisms between neighboring PorK units. These interactions involve the InsH domain, VR2, and VR4 regions (Fig. 4C). The first interaction site features residues E220, Y221, L224, Y162, and Y164 from the InsH domain, which interact with the loop formed by Y140, P143, V144, and T145 of the neighboring PorK’s InsH domain (Fig. 4C, upper). The second interaction site comprises a loop from InsH (L181, N182, and D184) engaging with residues Y406 and N436 from the neighboring PorK’s VR4 region (Fig. 4C, middle). The third interaction site combines residues R165, L169, and R170 from the InsH domain with the loop N388-K399 from the VR4 region, creating a pocket that accommodates the loop composed of P243, N244, A245, R246, and E248 from the VR2 region of the next PorK unit (Fig. 4C, bottom). These interaction sites, formed by the repurposed FGE-like fold and its insertion domains, are predominantly responsible for the formation of the PorK ring.

### Structure of PorN

**Fig 5:**
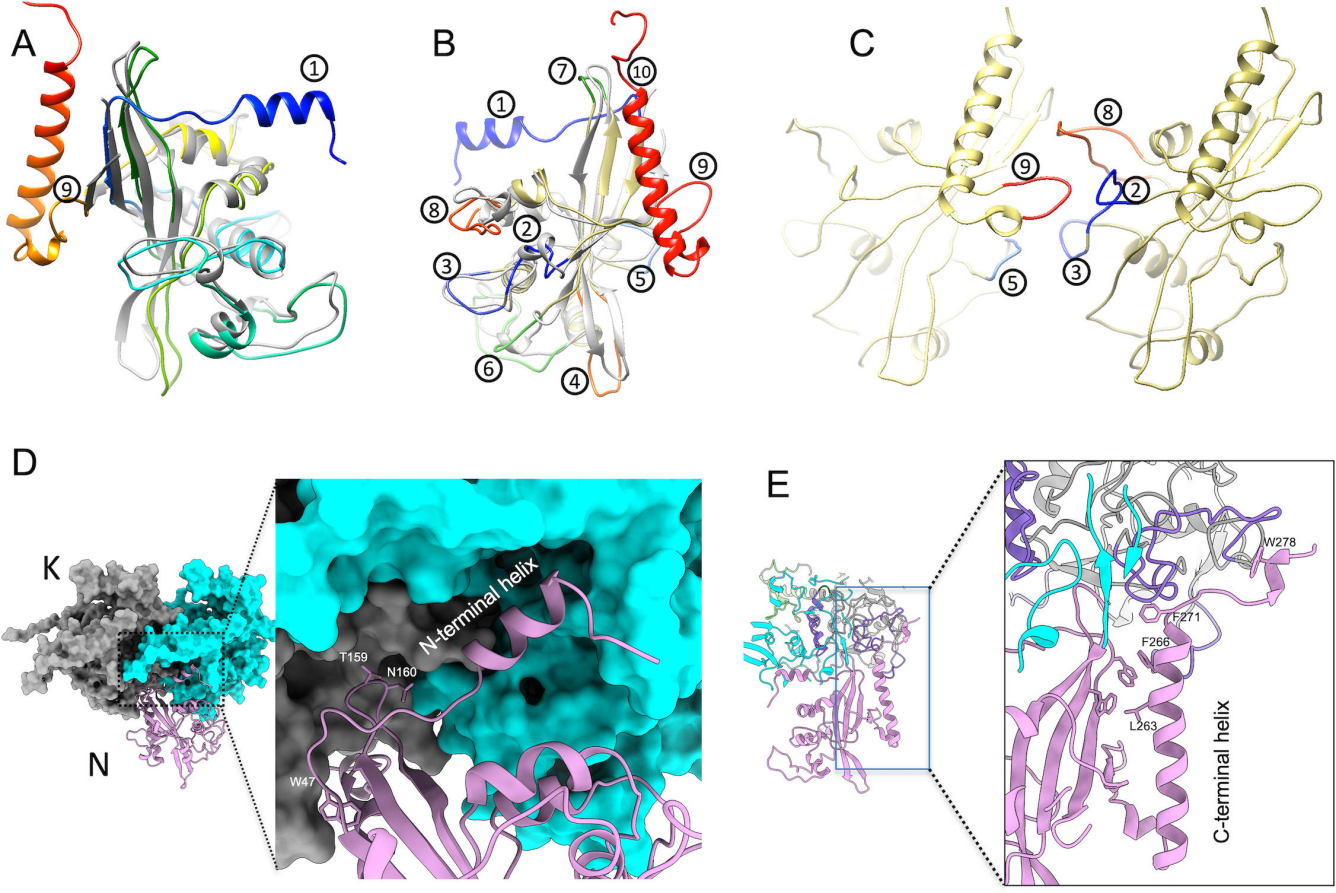
Interactions between PorN-PorN and PorK-PorN. (**A, B, and C**) PorN-PorN interactions. (**A and B**) Superimposition of the cryo-EM structure of PorN (this study, shown in rainbow) with the crystallographic structure (PDB ID: 7PVH [28]; colored gray) from different perspectives. Variable regions are labeled 1−10. (**C**) The interaction interface between two PorN subunits occurs between regions 9 and 5 of one unit and regions 2, 3, and 8 of the adjacent unit. (**D and E**) PorK-PorN interactions. (**D**) The extended N-terminal helix of PorN fits into a groove of PorK. (**E**) The extended C-terminal helix of PorN is stabilized by interaction with the VR3 variable region of PorK, shown in purple.

The cryo-EM structure of PorN closely matches the folding pattern observed in the crystal structure (PDB ID: 7PVH) (28), as shown in Fig. 5A and B. While the crystal structure resolved residues 45–239, our cryo-EM study extended this range to residues 25–281. This extended structure revealed a loop (L38-W47) and two additional terminal helices at the N- and C-termini, which were disordered in the crystal structure. The loop contributes two key residues, Q40 and W47, that interact strongly with R223 and F266 of the same PorN, respectively, stabilizing both the loop itself and the attachment of the C-terminal helix to the main body of PorN. The two additional helices (labeled 1 and 10 in Fig. 5B) and loop 7 interact with the top of the PorK structure, playing a crucial role in forming the PorKN ring. Compared to the crystal structure, loops 6 and 4 are displaced, suggesting conformational flexibility in this region of PorN, which facilitates PorM interaction with the PorKN ring. The region shared between the cryo-EM and crystal structures reveals an elongated antiparallel β-sheet, flanked on one side by five short α-helices. We identified eight distinct variable regions, with regions 2, 3, 5, 8, and 9 being particularly important for mediating interactions between adjacent PorN units, as shown in Fig. 5C.

PorN-PorN interactions emerge from two major conformational changes in regions 8 and 9. In variable region 8, a short helix of the crystal structure is disrupted and transformed into an extended loop that fits into a wedge-shaped space created by the splitting of two residues (E235 and N236) in region 9 from a β-strand in the main β-sheet. This extended loop 9 interlocks with the groove formed by regions 2, 3, and 8 of a neighboring PorN unit (Fig. 5C). However, these interactions appear relatively weak, as indicated by the low-density connecting adjacent PorN units. The findings suggest that the assembly of PorKN ring primarily relies on PorK-PorK and PorK-PorN interactions, with minimal contribution from direct PorN-PorN associations. That is, PorN units seem to anchor predominantly to the upper PorK structure.

### Intermolecular interactions of PorK and PorN

PorN interacts with two adjacent PorK units, engaging VR1, VR2, VR3, and VR5 from one unit and the VR4 region and InsH domain from the neighboring unit. This arrangement contributes to stabilizing the PorK-PorN interaction, with the two PorK units forming a pocket that accommodates PorN and integrates it into the PorK complex. The PorN interface exposed to the pocket includes two turning loops (residues N44-W47 and F156-D162) from the main β-sheet, along with additional N- and C-terminal helices.

The turning loop F156-D162 forms two hydrogen bonds: N160 with D391 of PorK and T159 with K419 of the adjacent PorK unit. The N44-W47 loop plays a role as well, with W47 forming a strong π-π interaction with F264 and a potential weaker π-π interaction with W47 in the VR1 region of PorK.

The extended N-terminal helix (residues 25−37), disordered in the PorN-only crystal structure, stabilizes within the PorK-PorN complex by fitting into a groove formed by the VR2 region, InsH domain, and VR4 region of an adjacent PorK subunit (Fig. 5D). Key residues R30, F34, and R37 anchor the helix through interactions with D198, Y398, and N244 of PorK, with the R30-D198 interaction being especially critical.

The extended C-terminal helix of PorN (residues P249-L270) is stabilized through interactions with the main β-sheet, including L263-W154 and F266-W47. Its distal loop (residues F271-Q283) anchors PorN to PorK via F271 interacting with E342 and W278 interacting with R328 and F334 of PorK (Fig. 5E). These interactions collectively ensure the stable integration of PorN within the PorK structure.

### Association of the PorLM motor to the PorKN ring

**Fig 6:**
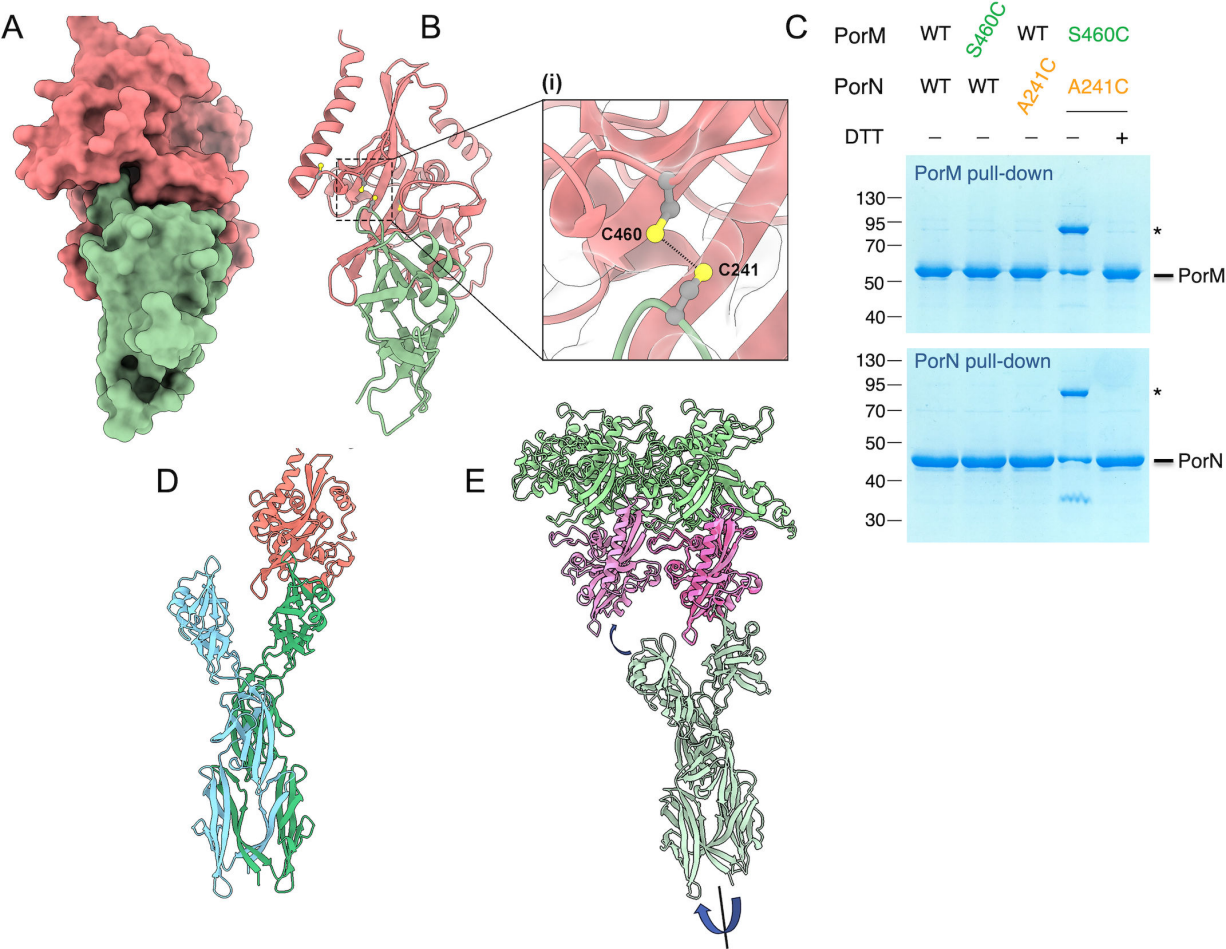
Structural model and *in vivo* validation of the PorM-PorN interaction. (**A and B**) Surface (**A**) and ribbon (**B**) representations of the AlphaFold2 structural model of PorN (red) with the PorM D4 domain (green). A magnification of a portion of the PorM-PorN interface, highlighting the side chains of the substituted residues: PorM S460 (green) and PorN A241 (orange) are shown as inset (i) in panel B. (**C**) Solubilized membrane extracts of *E. coli* cells producing VSV-G-tagged PorL, FLAG-tagged wild type (WT) or cysteine PorM variants, Strep-tagged WT or cysteine PorN variants, and 6× His-tagged PorK were subjected to pull-down on DYKD_4_K (top panel) or StrepTactin (bottom panel) resins. The eluate proteins were treated (+) or not (–) with DTT and subjected to 10% acrylamide SDS-PAGE and Coomassie Blue staining. The positions of PorM, PorN, and PorM-N complex (*) are indicated on the right. Molecular weight markers (in kDa) are indicated on the left. (**D**) AlphaFold2 model of the PorM D2-D4 dimer (blue and green) with one copy of PorN (red). (**E**) AlphaFold2 model of the PorM D2-D4 dimer/PorN plugged into the AlphaFold2 model of PorK_2_N_2_. PorM, PorN, and PorK are shown in light green, purple, and green, respectively.

*In situ* cryo-tomography imaging recently revealed that the PorKLMN trans-envelope complex is organized as a cage-like structure in which PorLM forms 18 pillars that are connected to the outer membrane-associated PorKN ring (10). PorM is a dimer composed of five regions: a transmembrane helix that is inserted within the PorL pentamer (22), a domain composed of a bundle of helices (D1), and three consecutive Ig-fold domains, D2-D4 (17). Interactions between the PorLM motor and the PorKN ring are mediated by contacts between the PorM C-terminal D4 domain and PorN (16, 17). First, we used AlphaFold2 to predict the complex between PorKN and PorM. The structure exhibited excellent metrics and resulted in a tight association. In agreement with experimental data (16, 17, 28), the model shows that the PorM D4 domain attaches onto the PorKN ring through interactions with PorN (Fig. 6A). Interestingly, PorM binds to loops 6 and 4 of PorN, which share different conformations in the cryo-EM and X-ray structures (Fig. 5A and B). To test this prediction *in vivo*, we positioned pairs of cysteine residues at the interface between PorM D4 and PorN, at locations susceptible to form a disulfide bridge, PorM S460 and PorN A241 (Fig. 6B). An ~95 kDa DTT-sensitive complex was pulled down by both PorM and PorN when PorM S460C and PorN A241C were co-produced (Fig. 6C). The mass of this complex is compatible with an association between PorM (theoretical mass, 56 kDa) and PorN (theoretical mass, 40 kDa), in agreement with mass spectrometry analyzes identifying both PorM and PorN.

To gain further insights into the PorM-PorN interaction, we modeled the association of a dimer of PorM D2-D4 to the PorK_2_N_2_ complex, which likely represents a repetition unit of the cage. This resulted in the partial destruction of the PorM dimer due to the attachment of each PorM D4 domain to adjacent PorN, resulting from a documented misbehavior of AlphaFold. We, therefore, predicted a complex between a PorM-D2-D4 dimer with PorN, which was then plugged onto PorK_2_N_2_. In the resulting structure, one D4 domain is engaged in a complex with one PorN, while the other D4 domain remains free (Fig. 6D). This suggests that the rotation of PorM could engage the free D4 domain with the next PorN (Fig. 6E) and hence triggers rotation of the ring (Movie S1).

## DISCUSSION

### FGE-like fold in PorK-like proteins functioning as a docking hub

We identified the FGE-like fold as a conserved structural motif in PorK of *P. gingivalis*, as well as in GldK and GldJ of *F. johnsoniae*, suggesting it could represent a shared feature across T9SS. Unlike the FGE, which activates sulfatases through cysteine-to-formylglycine conversion, the PorK FGE-like fold functions as a structural docking hub. Despite lacking the active-site cysteine residues characteristic of FGEs, PorK-like proteins utilize the FGE-like fold for two key roles: (i) integrating specific motifs internally for specialized functions and (ii) creating external structural pockets to accommodate PorN-like subunits.

**Fig 7:**
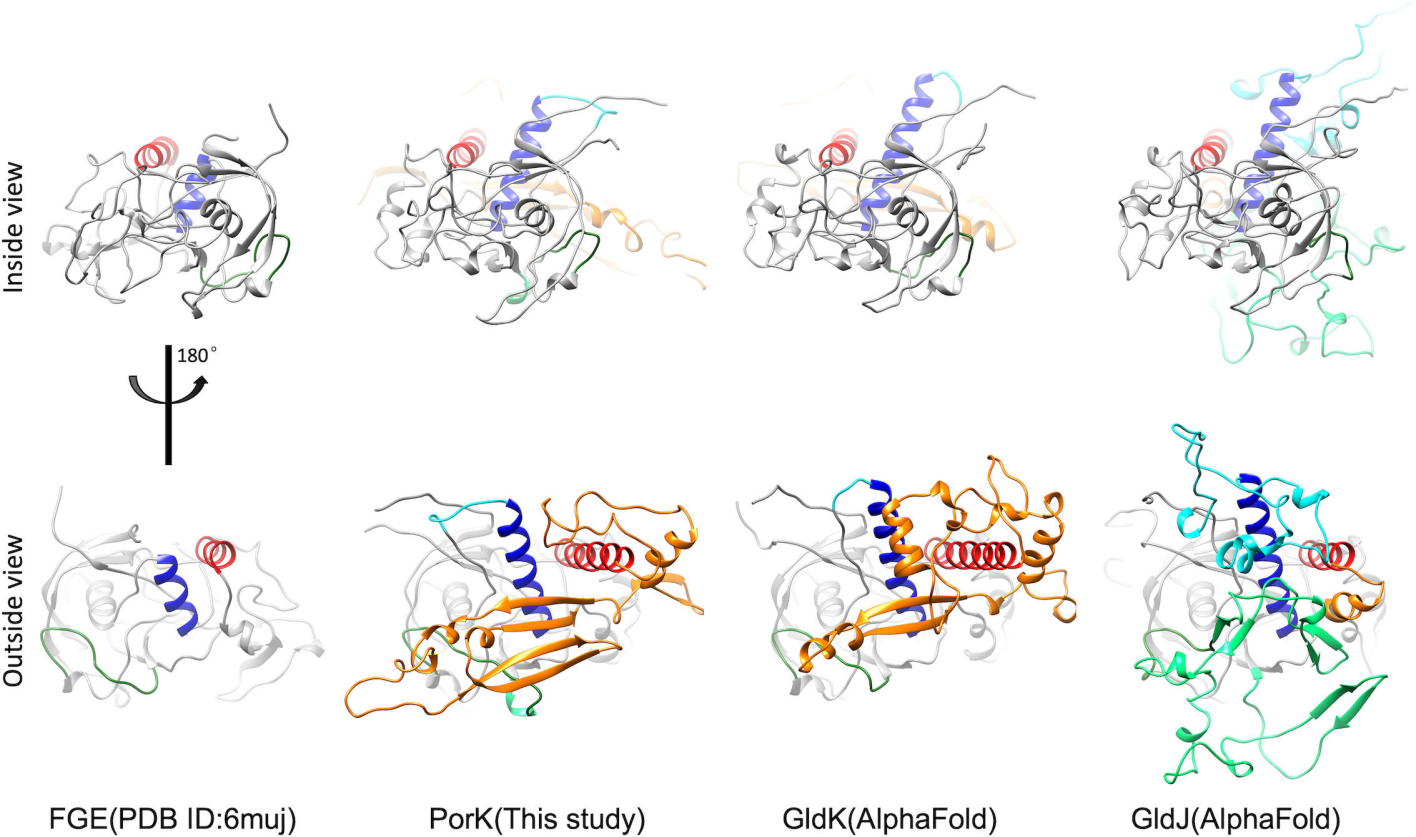
FGE-like fold in PorK-like proteins functioning as a docking hub. Three key insertion sites are highlighted: H-helix (red), V-helix (blue), and B-loop (dark green).

In analyzing the motif insertion hub regions, we identified three critical insertion sites by comparing the FGE structure with those of PorK, GldK, and GldJ (the latter two predicted by AlphaFold2). These sites, named the H-helix (red), V-helix (blue), and B-loop (dark green; Fig. 7), exhibit distinct structural adaptations in each protein. PorK features an extended H-helix with a prominent orange motif, facilitating adjacent PorK unit binding and controlling curvature to form a flexible ring structure. GldK, which shares structural similarity with PorK, also incorporates an orange motif, influencing curvature but potentially supporting a linear arrangement in *F. johnsoniae* as predicted by AlphaFold (Fig. S2). In contrast, GldJ retains the FGE-like fold with unique modifications; it shows minimal orange motif presence but significant insertions at the V-helix and B-loop. The arrangements of *P. gingivalis* PorK as a ring and of *F. johnsioniae* GldJK as a track reflect their two different functions: distributing effectors to Sov translocons for secretion and allowing the movement of adhesins along the cell body for gliding motility, respectively. These variations illustrate how structural adaptations of the FGE-like fold can dictate complex shapes and functions, highlighting its remarkable plasticity across diverse contexts.

Regarding the binding hubs for PorN, our findings reveal three variable regions within PorK’s FGE-like fold that play critical roles in facilitating interaction. Together with the orange motif, these regions form binding pockets accommodating the turning loops (residues P158-S161 and D43-W47) of PorN’s large β-sheet. They also establish charge interactions and anchoring points (PorK residues E342 and R328) for the C-terminal loop-helix (residues F271-M279) of PorN. Although the FGE-like domain of PorK is predicted to lack enzymatic activity, its variable regions act as interaction interfaces, akin to how FGE regions activate sulfatase substrates (29).

Without PorK, the terminal helices of PorN remain disordered, complicating protein crystallization. Consequently, the N- and C-terminal regions of PorN are unresolved in crystal structures (28). However, PorK’s FGE-like domain provides crucial hub ports for motif insertion and docking with PorN. These interactions enable PorK-PorK and PorK-PorN binding, which are essential for assembling the PorKN ring structure.

### The PorKN ring structure possesses inherent plasticity

Our structural analysis of the PorKN ring complex reveals several unexpected features that challenge previous models of its organization and assembly. A key finding is the inherent flexibility of the PorKN ring, which manifests in both symmetry variation and conformational heterogeneity.

We reconstructed the PorKN ring’s map using multiple symmetry numbers between 32 and 34, generating high-resolution maps that enable clear visualization of secondary structures. Two primary explanations address the observed symmetry number variation. The most compelling explanation focuses on the ring’s structural flexibility. Rather than a perfect circle, the ring demonstrates intrinsic curvature variations that allow for different symmetry representations. The 33-fold symmetry emerges as the predominant form, with some regions potentially showing 32- or 34-fold symmetries. This variability suggests that the ring structure possesses inherent plasticity, with the total copy number of PorK and PorN proteins likely centered around 33.

The focused refinement revealed nuanced structural characteristics, with central units resolving more clearly compared to the outer units. This approach not only confirmed the ring’s intrinsic flexibility but also provided unprecedented insight into its molecular architecture.

### Structural plasticity and substrate transport mechanism of the PorKN molecular machine

Molecular machines typically undergo conformational changes to facilitate substrate transport. The plasticity of the PorKN ring may provide valuable insights into how substrate translocation is coordinated across symmetrically distinct interfaces within the secretion system. Our recent cryo-ET study of the *P. gingivalis* T9SS revealed a unique supramolecular assembly comprising 18 translocation motors and eight export pores (14). In this study, the PorKN ring exhibits a 33-fold symmetry that starkly contrasts with the 18-fold symmetry of the underlying PorM complex and the eightfold symmetry of the Sov translocons positioned above. While symmetry mismatches are not uncommon in large molecular machines (30–32), the functional implications of these specific arrangements remain unclear. This structural disparity raises intriguing questions about how substrate translocation is managed across these distinct interfaces. The inherent flexibility of the PorKN ring, combined with its unique symmetry properties, may serve important functional roles in the secretion process by accommodating symmetry variation or enabling essential conformational changes during substrate translocation. Notably, the FGE-like domain of PorK provides critical docking sites for PorN binding and enables flexible movement within the complex. This aligns with our previous *in situ* cryo-electron tomography study, which revealed that PorKN can transition through three distinct conformational states during substrate transport, with angular shifts of 50°, 70°, and 90° relative to the outer membrane (14).

The substrate delivery pathway may be intricately linked to a special long helix in PorN (residues P249-L270) that is exposed on the ring’s inner surface and spans the entire PorN unit. This helix begins at the ring’s bottom and terminates with a crucial anchor point at PorK, where residue F271 interacts with PorK’s E342. Additional anchoring occurs through interactions between PorN’s W278 and PorK’s R328 and F334. These interactions, combined with the docking function of the FGE-like domain of PorK to PorN, create a sophisticated molecular mechanism that allows PorN units to “wobble” within the ring structure.

Several operating modes have been proposed for the T9SS. One model proposes that the energetic PorLM module drives these angled conformational changes, actively transporting effectors from PorM to the translocon. The peripheral arrangement of neighboring PorN units provides more radial flexibility than lateral movement, suggesting a “breathing” or “open/close” mechanism for substrate delivery, compatible with this first model (Fig. S3). In the second model, the PorLM motor energizes rotation of the entire PorKN ring or only of the PorN subring relative to the PorK subring. Based on the PorKN structure presented here, the PorN-PorK subring rotation mechanism appears unlikely. Such a scenario would require detaching N- and C-terminal helices from PorK, potentially causing the entire PorKN ring to disassemble. This would introduce an energetically inefficient re-assembly process that seems unnecessary for substrate transport. The structural arrangement of PorKN allows for dynamic movement of the whole ring while maintaining the overall integrity of the molecular machine, enabling efficient and controlled substrate delivery through the T9SS (Fig. S3).

Our structural predictions of the association of PorM pillars with the PorKN ring suggest that PorM binds in the cavity between two neighboring PorN copies, involving a single D4 domain of the PorM dimer, while the second D4 domain is free. One can speculate that the chemical energy provided by proton-motive force is transduced to the ring structure via PorM through a rotary mechanism (25, 33). Indeed, cryo-ET imaging and limited proteolysis data have shown that PorM changes conformation depending on the pmf (14, 21). This conformational change could be conveyed to PorN, either impacting the angled conformation of the ring or by inducing rotation of the ring. Future studies will be crucial for understanding how these structural features and structural transitions contribute to the mechanism of protein secretion through this complex molecular machinery.

## MATERIALS AND METHODS

### *P. gingivalis* culture

*P. gingivalis* 33277 and W83 and their isogenic mutants were cultured anaerobically at 37°C in Trypticase soy broth supplemented with 1 mg/mL yeast extract, 5 µg/mL hemin, and 1 µg/mL menadione. When appropriate, erythromycin (10 µg/mL) was added to the medium.

### Plasmid and strain constructions

To generate a strain expressing a Strep-tagged version of PorK, a gene fusion consisting of the promoter region 492 bp upstream of the porP gene fused to the coding region of porK with a C-terminal twin strep tag (SAWSHPQFEKGGGSGGGSGGGAWSHPQFEK) was synthesized (Azenta). The resulting gene fusion was cloned into pT-COW. The plasmid, pPorK-Strep, was transferred into the *P. gingivalis* ΔwbaP strain via conjugation with *E. coli* (S17-1 strain) as described (34). pRSF-_VSVG_PorL-_FLAG_PorM and pCDF-PorK_His_-PorN_ST_ vectors were engineered by restriction-free cloning as described (16). All PCRs were performed with the Q5 High-Fidelity DNA polymerase (NEB). The pRSF-_VSVG_PorL-_FLAG_PorM vector was constructed by a two-step cloning procedure in which porL (fused to a VSVG tag sequence at the N-terminus, amplified from *P. gingivalis* genomic DNA) was first inserted. A PCR fragment comprising a ribosome binding site (RBS) and porM fused to an N-terminal FLAG tag was inserted in tandem with VSVG-porL. The pCDF-PorK_His_-PorN_ST_ was engineered similarly, except that PorK and PorN were amplified from plasmids pIBA4-PorK (16) and pIBA4-PorN (16), respectively. These two plasmids bear PorK and PorN fused to *E. coli* OmpA signal sequence for proper export to the periplasm in *E. coli*. Substitutions were introduced by Quick-Change site-directed mutagenesis using complementary oligonucleotides bearing the desired mutations. The sequences of all insertions and mutations were confirmed by DNA sequencing (Eurofins Genomics).

### Purification of PorKN complexes

*P. gingivalis* cells were resuspended in lysis buffer (50 mM Tris-HCl, pH 7.5, 150 mM NaCl, 0.5 M sucrose, 5 mM MgCl_2_, 1% n-dodecyl-β-D-maltopyranoside [DDM], cOmplete protease inhibitor cocktail [Roche], and benzonase) and incubated in ice for 45 min. Unbroken cell debris was removed by centrifugation at 10,000 × *g* for 25 min at 4°C. The supernatant was centrifuged at 142,000 × *g* for 40 min at 10°C, and the pellet was resuspended in 50 mM Tris-HCl (pH 7.5), 500 mM NaCl, 1% DDM, 5 mM EDTA, 0.5 mg/mL lysozyme, and cOmplete protease inhibitor cocktail. The sample was incubated at 37°C for 15 min and centrifuged again at 142,000 × *g* for 40 min. The pellet was resuspended in 0.5% Triton X-100, 10 mM Tris-HCl (pH 7.5), 500 mM NaCl, and 5 mM EDTA. The insoluble fraction was removed by centrifugation at 10,000 × *g* at 4°C for 10 min. The supernatant was transferred to a Strep-Tactin column. The column was washed five times with 1 column volume (CV) buffer W (100 mM Tris-HCl [pH 8.0], 150 mM NaCl, 1 mM EDTA, and 0.5% Triton X-100). For elution, buffer E (buffer W containing 5 mM D-desthiobiotin) was added to the column, and proteins were collected in 0.5 CV fractions. The fractions were concentrated by ultracentrifugation at 310,000 × *g*, followed by western blot analysis.

### Cryo-EM sample preparation and data collection parameters

The purified PorKN complex (~3.0 µL) was applied onto a glow-discharged Lacey 300 mesh Au grid with ultrathin carbon film. The sample was allowed to absorb to the grid for 1 min at 22°C and 100% humidity in a Vitrobot (FEI), before blotting for 4 s at −7 force and being plunge-frozen in liquid ethane. The frozen grids were stored in liquid nitrogen until imaging. The data were collected using an FEI Titan Krios transmission electron microscope, operated at 300 kV, and equipped with Gatan GIF Quantum energy filter. The micrographs were acquired with EPU software (FEI), recorded on a K2 Summit direct electron detector (Gatan) operated in counting mode with a physical pixel size of 1.07 Å per pixel. The detector was placed at the end of a GIF Quantum energy filter operated in zero-energy-loss mode with a slit width of 20 eV. The total exposure time was 7 s, and intermediate frames were recorded every 0.2 s, giving an accumulated dose of ~40 e^−^/Å^2^ (2) and a total of 35 frames per image. The defocus range used was from −0.8 µm to −2.8 µm.

### Cryo-EM data collection, image processing, and model building

After our preliminary studies revealed coarse information about image quality, particle size, and distribution, we refined our purification process and initiated large-scale data collection. To address the preferred orientation issue, as most particles were predominantly distributed at 0° (top view), we employed a tilting strategy by adjusting the specimen stage. This approach resulted in the collection of 2,000, 2,300, and 1,800 images at tilt angles of 0°, 15°, and 25°, respectively.

Data processing was carried out using cryoSparc software (Fig. S4). Motion correction was applied to each image stack, aligning the 35 drifted frames and merging them into a single high-quality image. contrast transfer function (CTF) parameters were subsequently determined using the patch CTF module. Targeted ring particle images were automatically picked based on their approximate diameter. These particles were further evaluated to retain only those with high-quality CTF (resolution better than 4.5 Å), while excluding particles located outside grid holes. Finally, 2D classification was applied to remove suboptimal images, resulting in approximately 42,000 high-quality particles with a box size of 720 pixels.

Using the ab initio reconstruction module of CryoSPARC, we classified particles into three categories: 10,000 single-ring particles, 27,000 double-ring particles, and 5,000 uncertain structures. We tested symmetries ranging from 31- to 35-fold during refinement for both single- and double-ring data sets, with 31- and 35-fold symmetries determined less likely. Single-ring structures achieved resolutions of 8.4 Å, 7.4 Å, and 8.6 Å with 32-, 33-, and 34-fold symmetries, respectively, while double-ring structures reached 6.1 Å, 5.6 Å, and 6.0 Å with the same symmetries. These results indicated 33-fold symmetry as the most plausible configuration.

Variations in 32- to 34-fold symmetries were attributed either to rings containing different numbers of unit copies or to the ring’s inherent plasticity, where different regions exhibit variable curvatures. Regardless of the cause, localized segments within a ring displayed higher structural similarity than that observed between different rings. Consequently, we performed local refinement to enhance the map’s resolution for the double-ring structure, given its superior stability compared to the single-ring structure.

Using particle orientations from our previous 33-fold symmetry-imposed reconstruction of the complete ring particles, we expanded each particle’s orientation into 33 symmetrized orientations across the entire data set. We then determined the centers for each of these symmetrized units and re-boxed each ring particle into 33 sub-particles centered at individual ring units, generating a total of 1,153,755 sub-particles with a box size of 300 pixels. We performed focused refinement on these sub-particles that locally refined their orientations with C1 symmetry to a resolution of 4.3 Å, and we further improved the resolution to 3.2 Å by re-boxing the sub-particles with a smaller box size of 200 pixels (Fig. S4).

The 3.2 Å map provided sufficient resolution to segment components and build structural models for PorK and PorN. Due to the ring’s plasticity under C1 symmetry refinement, the map exhibited non-uniform resolution with better-resolved central regions. We used these well-defined central densities to construct models for PorK and PorN using Coot (35), which was also used for validation. The constructed models were then expanded to fit the two side densities around the central regions, allowing us to create structural models for three consecutive units within the PorKN ring.

### Structure prediction

Structure predictions were conducted using AlphaFold2 (36), using access to the Institut du Développement et des Ressources en Informatique Scientifique (IDRIS) Jean-Zay multi-graphics processing unit (GPU) server equipped with Nvidia A100 GPUs and 80 GB RAM. Reliability of the prediction and of the interaction was measured by the predicted local distance difference test (pLDDT) and predicted aligned error (PAE) outputs. pLDDT is a per-residue measure of local confidence scaled from 1 to 100, and PAE is a measure of how confident AlphaFold is in the relative position of two residues within the predicted structure. All predictions that did not satisfy a >80% pLDDT and PAE criteria were discarded. Visual inspection and analysis of the structures were carried out using either Coot (35) or ChimeraX (37). Predicted structure files, as well as pLDDT and PAE files, have been deposited with Zenodo (38).

### Disulfide cross-linking assay

*E. coli* BL21(DE3) cells carrying plasmids pRSF-_VSV-G_PorL -_FL_PorM (producing N-terminally VSV-G-tagged PorL and N-terminally FLAG-tagged PorM) and pCDF-PorK_His_-PorN_ST_ (producing C-terminally 6× His-tagged PorK and C-terminally Strep-tagged PorN), or their cysteine variants, were grown to an absorbance at λ = 600 nm (*A*_600_) of 0.4, and gene expression from pRSF/pCDF vectors was induced for 3 hours at 30°C with 0.1 mM of IPTG. About 4 × 10^11^ cells were then harvested by centrifugation and resuspended in 20 mL of ice-cold buffer A (20 mM Tris-HCl [pH 8], 150 mM NaCl, cOmplete protease inhibitor cocktail [Roche]) supplemented with 2.5 mM of *N-*ethylmaleimide (NEM) to block all free thiol groups and avoid unspecific post-lysis disulfide-bond formation. After 20 min on ice, cells were broken at 1,000 psi using an EmulsiFlex-C5 (Avestin), and unbroken cells were discarded by centrifugation. Cell envelopes were obtained by ultracentrifugation at 90,000 × *g* for 45 min and resuspended in 0.5 mL of buffer A supplemented with 2.5 mM NEM and 1% of SDS to solubilize proteins and break uncovalently bound complexes. After 1 hour of incubation with rotation, 8 mL of buffer A supplemented with 0.8% of DDM (Anatrace) was added. After 30 min rotation, the insolubilized material was discarded by ultracentrifugation, and the cell lysate was further incubated for one hour with 150 µL of nickel (Protino Ni-NTA Agarose, Nagel), Streptag (Step-Tactin Sepharose, IBA), or FLAG (anti-DYKD_4_K affinity resin Ultra-Link, Pierce) resins. Resins were washed three times with 30 volumes of buffer A supplemented with 0.8% of DDM and twice with 30 volumes of buffer A supplemented with 0.1% of DDM, before three consecutive elutions with one volume of buffer A supplemented with 0.1% of DDM and 250 mM of imidazole, 2.5 mM of desthiobiotin, or 3× DYKDDDK peptides (Pierce). The three elutions were combined, mixed with 4× Laemmli loading buffer, supplemented or not with 100 mM of DTT, and boiled for 10 min before protein separation by SDS-PAGE on 10% acrylamide gels. Proteins were stained with Coomassie (InstantBlue Coomassie Protein Stain, Abcam).

## Data Availability

Cryo-electron microscopy density maps and atomic coordinate data for the T9SS PorKN ring structures have been deposited in the Electron Microscopy Data Bank (EMDB) and Protein Data Bank (PDB). Single-ring PorKN reconstructions are available under accession codes EMD-70849 (C33), EMD-70850 (C34), and EMD-70851 (C32). Double-ring PorKN reconstructions are deposited as EMD-70853 (C33), EMD-70854 (C34), and EMD-70856 (C32). The segmented high-resolution C1 reconstruction is available as EMD-70857, with the corresponding atomic model deposited in PDB as 9OTS. The authors declare that all other data supporting the findings of this study are available within the paper and its supplemental materials, except the predicted structure files, pLDDT, and PAE values, which have been made available at Zenodo https://doi.org/10.5281/zenodo.15235176 (38).

## SUPPLEMENTAL MATERIAL

Supplemental Figures mbio.01799-25-s0001.docx Figures S1 to S4.

Movie S1 mbio.01799-25-s0002.mp4 Prediction of the movement of a rotating PorM dimer along the PorK-PorN ring.

ASM does not own the copyrights to Supplemental Material that may be linked to, or accessed through, an article. The authors have granted ASM a non-exclusive, world-wide license to publish the Supplemental Material files. Please contact the corresponding author directly for reuse.
