# Supplementary material for: Structure of the T9SS PorKN ring complex reveals conformational plasticity based on the repurposed FGE fold
Source: mBio. 2025 Aug 7;16(9):e01799-25. doi: 10.1128/mbio.01799-25 (PMC12421860; doi:10.1128/mbio.01799-25)
Supplement: Supplemental Figures — Figures S1 to S4. [file mbio.01799-25-s0001.docx]

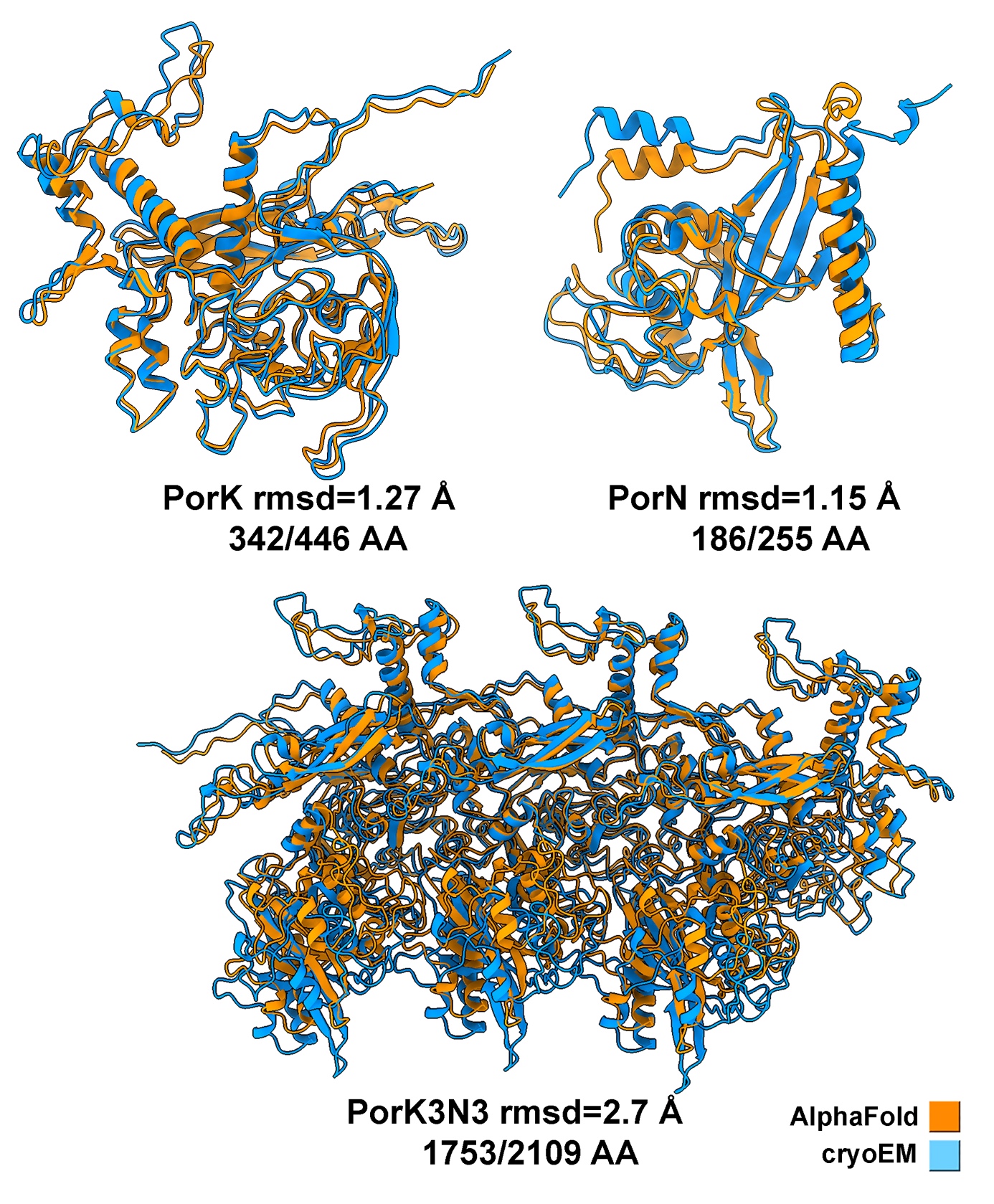


**Supplementary Figure S1. Comparison between the PorK (A), PorN (B) and PorK_3_N_3_ (C) structures predicted by AlphaFold2 (orange) and determined by cryoEM (blue).** For each comparison, the r.m.s.d. is indicated.


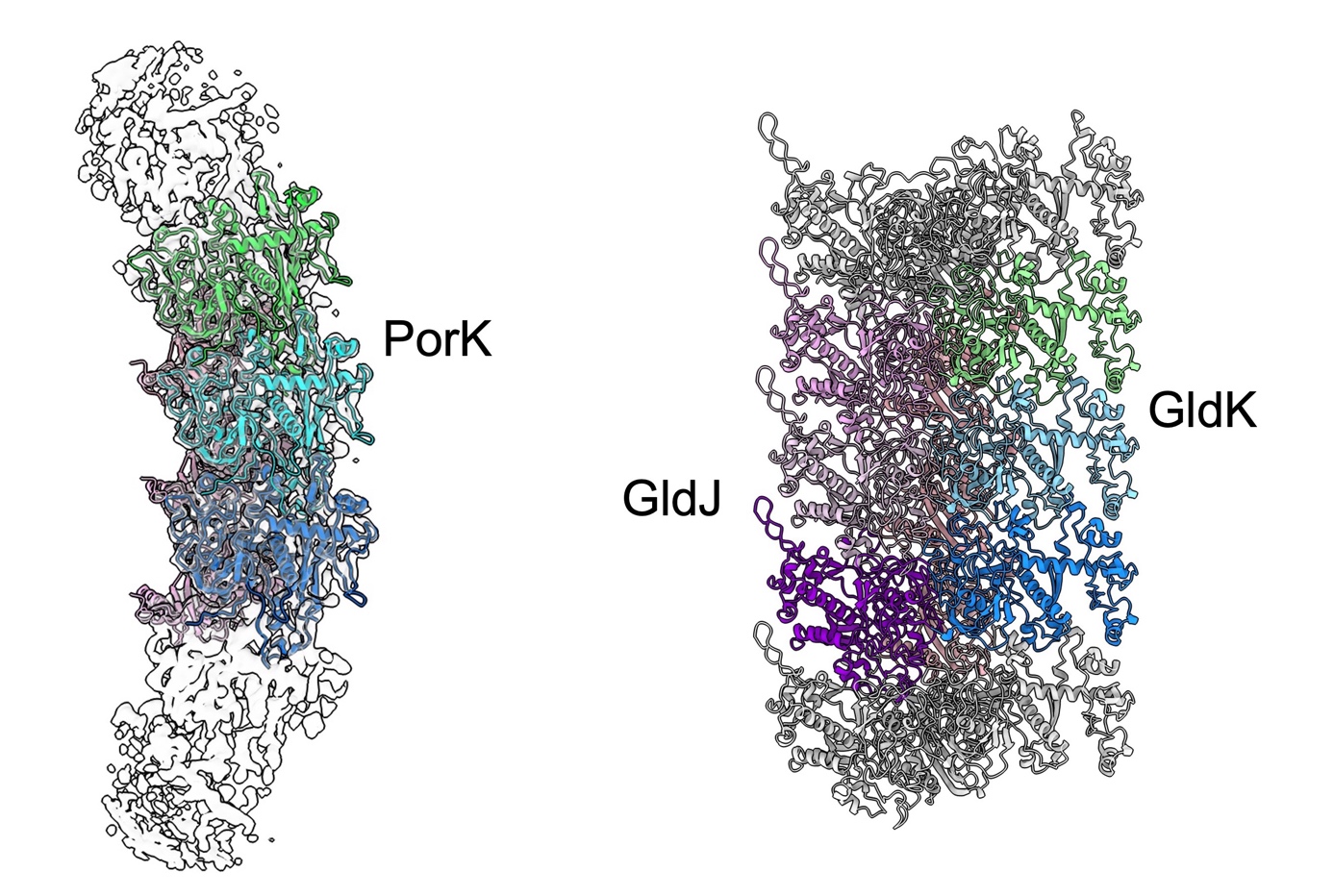


**Supplementary Figure S2. PorK curvature and potential linear arrangement of GldK.** Three consecutive PorK proteins are shown on the left. The GldK/J model was predicted by AlphaFold. Three consecutive GldK/J proteins are shown on the right.


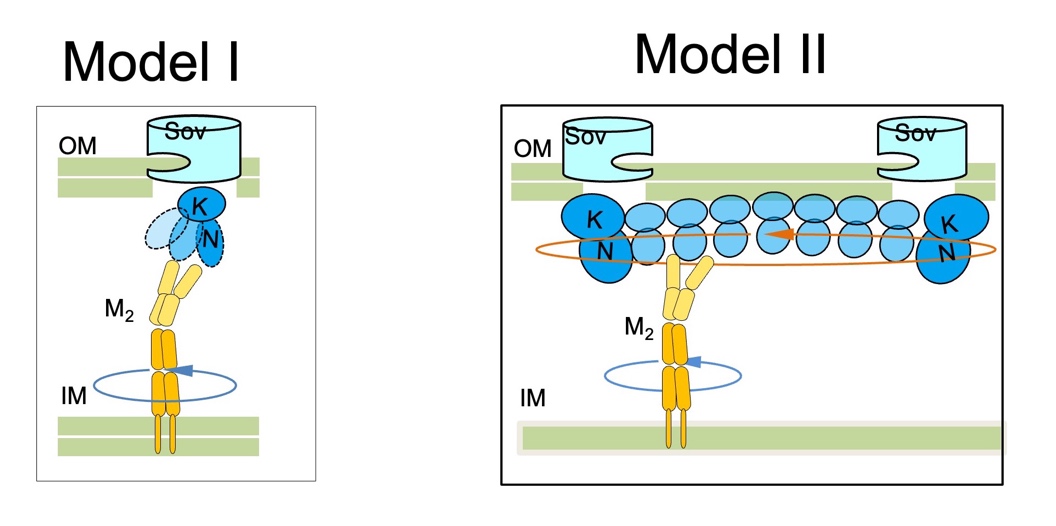


**Supplementary Figure S3.** Schematic of the *P. gingivalis* T9SS working model: I) PorLM module drives these angled conformational changes of PorKN, actively transporting effectors from PorM to the translocon. II) PorLM motor energizes rotation of the entire PorKN ring, enabling efficient substrate delivery through the T9SS.


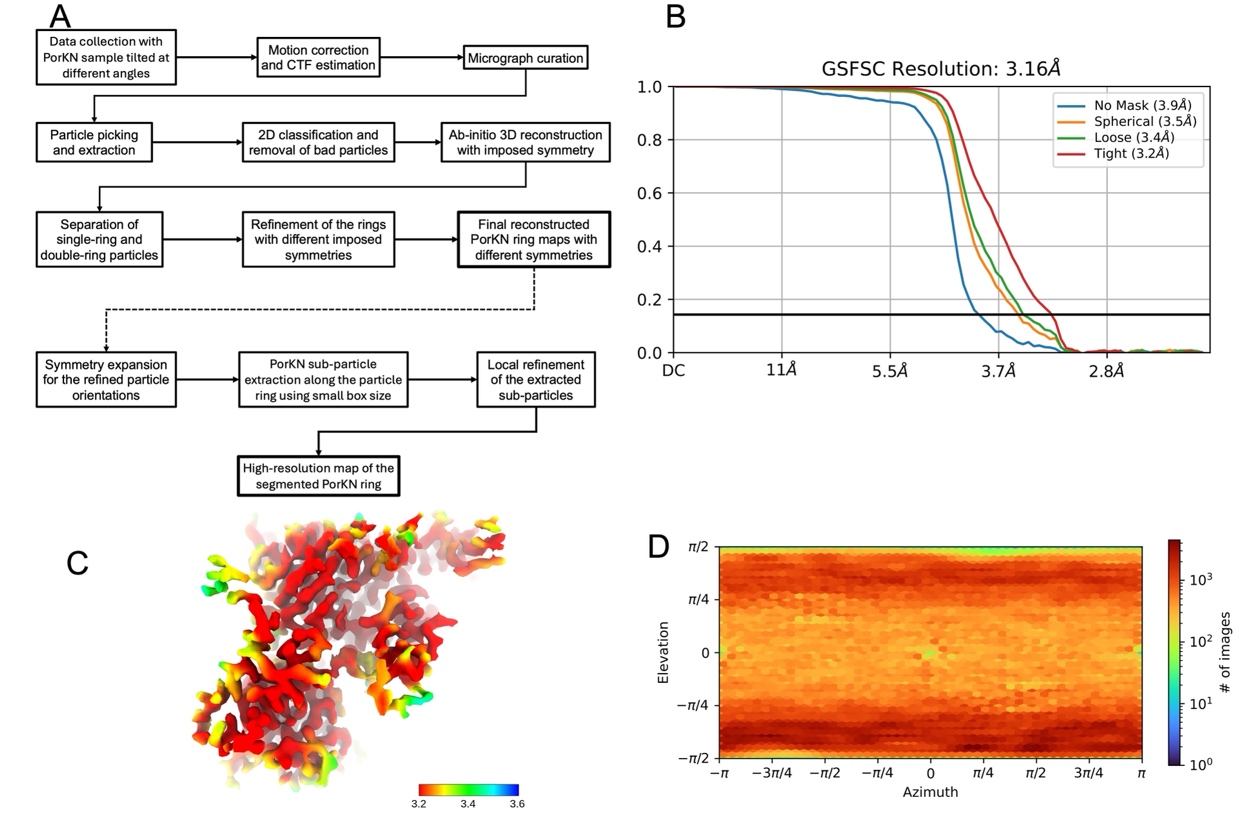


**Supplementary Figure S4** (A) Flowchart of CryoSPARC processing pipeline for PorKN particle images. (B) Fourier Shell Correlation (FSC) resolution curves for final maps from CryoSPARC refinement. Resolutions were determined using FSCs at the 0.143 cutoff. (C) Local resolution map calculated by CryoSPARC's local resolution estimation. (D) Heatmap showing orientation distribution of final particles.

**Supplemental Movie 1.** Prediction of the movement of a rotating PorM dimer along the PorK-PorN ring.
